# 3D electron microscopy reveals novel ultrastructural changes in the diabetic retinal neurovascular unit

**DOI:** 10.1007/s00125-025-06554-9

**Published:** 2025-10-02

**Authors:** Mona J. Albargothy, Evan P. Troendle, Ross Laws, Peter Barabas, David H. W. Steel, Michael J. Taggart, Tim M. Curtis

**Affiliations:** 1 Biosciences Institute, Newcastle University, Newcastle upon Tyne, UK; 2 Wellcome-Wolfson Institute for Experimental Medicine, Queen’s University Belfast, Belfast, UK

**Keywords:** 3D ultrastructural changes, Basement membrane detachment, Endothelial tubules, Glial cell retraction, Inner blood–retina barrier, Peg-and-socket formations, Pericyte–endothelial cell interactions, Retinal capillary stability, Retinal neurovascular unit, Serial block-face scanning electron microscopy

## Abstract

**Aims/hypothesis:**

This study used serial block-face scanning electron microscopy (SBF-SEM), a nanoscale imaging technique in x-y-z planes, to investigate 3D ultrastructural changes in the retinal neurovascular unit (NVU) associated with diabetes. We hypothesised that this approach would reveal previously uncharacterised pathological alterations that contribute to the development of diabetic retinal disease (DRD).

**Methods:**

Retinas from male diabetic and non-diabetic mice, as well as from human male donors with and without diabetes, were prepared for SBF-SEM imaging. Retinal tissue was microdissected, fixed and embedded for serial sectioning and 3D reconstruction. Ultrastructural analysis of the NVU was performed in capillary regions exclusively within the superficial vascular plexus of both mouse and human retinas. Image stacks were processed using Microscopy Image Browser for contrast normalisation and segmentation, with 3D visualisation performed in Amira software. Quantitative analyses were conducted on pericyte–endothelial cell peg-and-socket formations, cell–basement membrane (BM) interactions, endothelial tubule formation and vascular BM thickness.

**Results:**

SBF-SEM revealed novel 3D ultrastructural changes in the retinal NVU of diabetic mice and humans, including: (1) partial detachment and reduced frequency of pericyte–endothelium peg-and-socket formations (*p*<0.05–0.001); (2) localised detachment of endothelial cells and pericytes from the vascular BM (*p*<0.05–0.01), along with macroglial cell retraction from the outer vascular BM; and (3) increased formation of endothelial tubules (*p*<0.01–0.001). These changes were observed in the absence of any obvious vascular BM thickening, as no significant differences in mean or maximum BM thickness were found between diabetic and non-diabetic retinal capillaries analysed in this study.

**Conclusions/interpretation:**

This study provides new insights into the early ultrastructural changes in the retinal NVU in DRD, offering a basis for a better understanding of the pathological processes that contribute to the development of this disease.

**Data availability:**

Links to all raw image stacks analysed in this article are available at https://doi.org/10.5281/zenodo.15210333. The MATLAB vascular BM thickness measurement script is available at the GitHub link https://github.com/Curtis-WWIEM/BM_thickness.

**Graphical Abstract:**

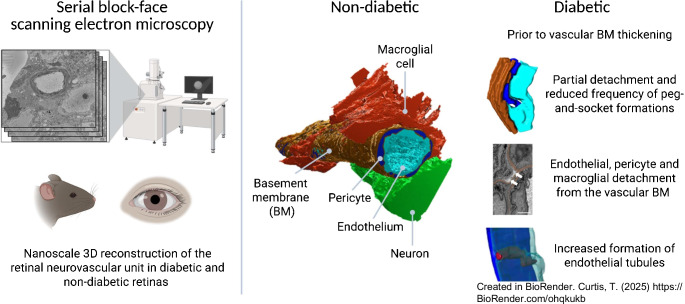

**Supplementary Information:**

The online version of this article (10.1007/s00125-025-06554-9) contains peer-reviewed but unedited supplementary material.


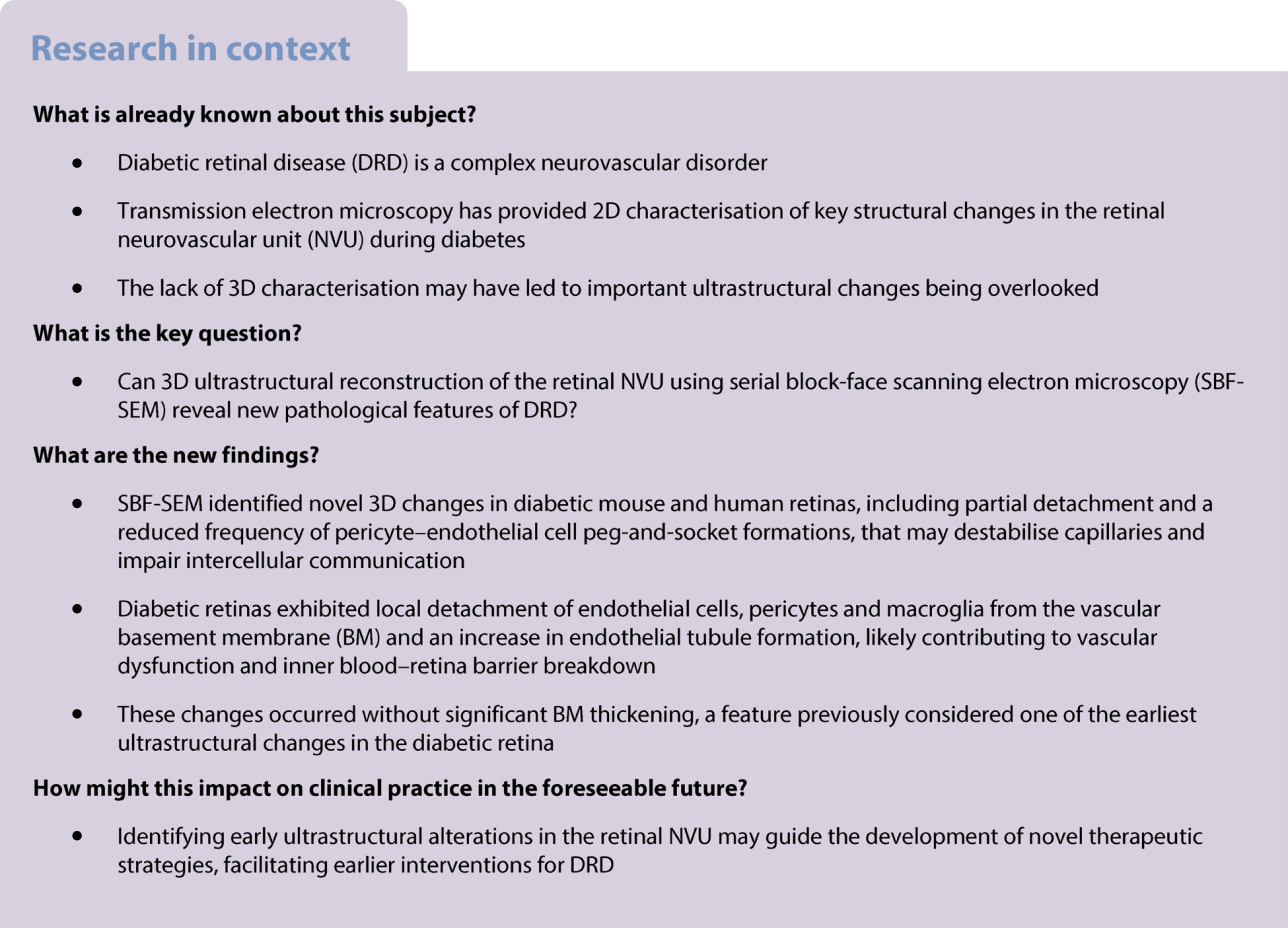


## Introduction

Electron microscopy has been instrumental in advancing our understanding of diabetic retinal disease (DRD), providing detailed insights into key structural changes in the retina associated with disease onset and progression. Early studies from the mid-20th century identified hallmark features of DRD, including capillary basement membrane (BM) thickening, pericyte loss, microaneurysm formation and retinal capillary occlusion [1]. More recent research has also highlighted the importance of neural changes, such as gliosis, synaptic abnormalities and neuronal degeneration, reshaping our view of DRD as a disease of the retinal neurovascular unit (NVU) rather than being solely a microvascular condition [2].

Historically, transmission electron microscopy (TEM) has been the primary technique for analysing ultrastructural changes in DRD. While TEM has enabled high-resolution 2D (x-y) analysis of the retinal NVU during diabetes, reliance on this method may have led to critical ultrastructural alterations being overlooked, changes that may only become evident through examination of the 3D (x-y-z) organisation and cellular interactions within the retinal NVU.

To address this limitation, we recently developed a pipeline for the 3D nanoscale reconstruction and characterisation of the healthy murine retinal NVU using serial block-face scanning electron microscopy (SBF-SEM) [3]. In the present study, we applied this approach to investigate 3D ultrastructural alterations in the retinal NVU in a mouse model of diabetes, alongside comparative analysis of human diabetic retinal samples, focusing on capillary regions from the superficial vascular plexus in both species. Our aim was to identify and characterise diabetes-associated changes in NVU architecture that may contribute to the pathophysiology of DRD.

## Methods

For detailed methods, please refer to electronic supplementary material (ESM) Methods.

### Diabetic mouse and human samples

All animal procedures were approved by Queen’s University Belfast AWERB and conducted under the UK Animals (Scientific Procedures) Act 1986. C57BL/6J mice were injected intraperitoneally with streptozocin (STZ; 50 mg/kg) for five consecutive days. Diabetes (blood glucose ≥18 mmol/l) was confirmed 1 week later. Control mice were treated with citrate buffer alone. Mice were euthanised by CO_2_ asphyxiation and cervical dislocation 6 months after diabetes induction.

Human studies were approved by the Newcastle University Ethics Committee and conducted in accordance with the Declaration of Helsinki. Eye samples were obtained from individuals undergoing exenteration for facial or sinus tumours: a 58-year-old man without diabetes; and a 67-year-old man with type 2 diabetes of 10 years duration.

### Sample preparation, image collection and processing

Mouse and human retinas were microdissected, fixed in 2% wt/vol. glutaraldehyde and embedded in resin for SBF-SEM imaging. Resin-embedded retinal tissue was imaged using a Zeiss Sigma SEM Gatan 3 View system at a resolution of 6 nm/pixel, capturing 130–300 serial sections per image set (each section representing 100 nm in the z-axis). Image sets from 18 mouse retinal NVUs (three each from three diabetic and three non-diabetic mice) and six human parafoveal retinal NVUs (three from each retina), all located in the superficial vascular plexus, were processed for contrast normalisation, alignment and segmentation using Microscopy Image Browser. 3D visualisation was performed using Amira software.

### Image analysis

Specific NVU features, including pericyte–endothelial cell peg-and-socket formations, cell–BM detachment and endothelial tubules, were identified and analysed based on defined inclusion/exclusion criteria (see ESM Methods). Vascular BM thickness across the image stacks was measured using a custom MATLAB script (see ESM Methods).

## Statistics

Data are presented as mean ± SEM with individual data points shown. Comparisons were made using unpaired two-tailed Student’s *t* tests or Mann–Whitney *U* tests, with *p*<0.05 considered significant.

## Results

Visual inspection of our SBF-SEM image stacks revealed several changes in the retinal NVU of diabetic mice and a human diabetic sample, none of which have been previously described or characterised in 3D. These changes were further analysed.

### Peg-and-socket formations

**Fig. 1:**
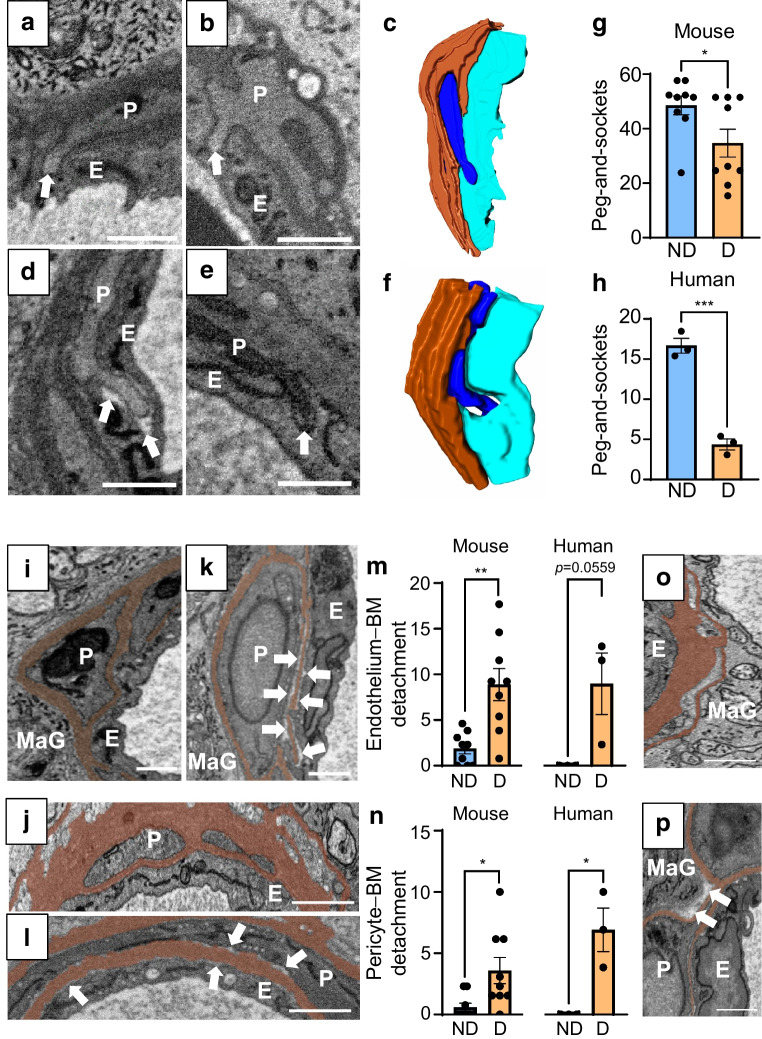
Disruption of peg-and-socket formations and cell–BM interactions in diabetic retinal capillaries. (**a**, **b**) Electron micrographs of retinal capillaries from a non-diabetic mouse (**a**) and an individual without diabetes (**b**), showing close alignment of pericyte–endothelial cell peg-and-socket plasma membranes (white arrows indicate pericyte pegs). Scale bars, 1 µm. (**c**) 3D reconstruction of the peg-and-socket formation in (**a**). Pericyte shown in blue, endothelium in aqua and BM in brown. (**d**, **e**) Electron micrographs of retinal capillaries from a diabetic mouse (**d**) and an individual with diabetes (**e**), showing disrupted peg-and-socket formations with electron-lucent gaps (white arrows). Scale bars, 1 µm. (**f**) 3D reconstruction of the disrupted connection in (**d**). Pericyte shown in blue, endothelium in aqua and BM in brown. (**g**, **h**) Quantification of peg-and-socket formations per 10 µm retinal capillary depth in non-diabetic and diabetic mice (*n*=3 mice, nine capillaries per group) and human donors (*n*=1 donor, three capillaries per group). *p<0.05, ***p<0.001. (**i**, **j**) Electron micrographs of retinal capillaries from a non-diabetic mouse (**i**) and an individual without diabetes (**j**), showing endothelial cells and pericytes in close contact with the vascular BM (segmented in brown). Scale bars, 1 µm. (**k**, **l**) Electron micrographs of retinal capillaries from a diabetic mouse (**k**) and an individual with diabetes (**l**) Scale bars, 1 µm. Electron-lucent gaps (white arrows) indicate localised detachment from the BM. (**m**, **n**) Quantification of endothelial (**m**) and pericyte (**n**) detachment frequency per 10 µm capillary depth in non-diabetic and diabetic mice (*n*=3 mice, nine capillaries per group) and human donors (*n*=1 donor, three capillaries per group). *p<0.05, **p<0.01. (**o**, **p**) Electron micrographs of retinal capillaries from individuals without (**o**) and with (**p**) diabetes, highlighting macroglial cell retraction from the outer vascular BM in diabetes (white arrows). Scale bars, 1 µm. D, diabetes; E, endothelial cell; MaG, macroglia; ND, no diabetes; P, pericyte

Our previous work has shown that peg-and-socket formations are key features of pericyte–endothelial cell interactions in retinal capillaries [3]. These structures are believed to anchor the two cell types together and serve as sites for the enrichment of signalling proteins, facilitating cellular crosstalk [4, 5]. In non-diabetic capillaries from both mice and humans, we observed close alignment of the peg-and-socket plasma membranes (Fig. 1a–c). In contrast, in retinas from STZ-induced diabetic mice and an individual with diabetes, many of these formations displayed enlarged electron-lucent socket spaces, indicating partial detachment of the pegs from the sockets (Fig. 1d–f). Additionally, analysis of peg-and-socket distribution and abundance revealed a significant reduction in their frequency in retinal capillaries of mice and humans with diabetes (Fig. 1g, h and ESM Fig. 1), with the decrease being particularly pronounced in the human retinal capillaries.

### Cell–BM interactions

Another notable feature observed in our SBF-SEM imaging was localised cell detachment from the vascular BM in diabetic retinal capillaries. In non-diabetic retinas, endothelial cells, pericytes and macroglia maintained close contact with the BM along its entire length (Fig. 1i, j). In contrast, diabetic mouse and human samples displayed localised regions with electron-lucent gaps between the cell plasma membranes and the BM, indicating cell detachment (Fig. 1k, l). Quantification of detachment sites confirmed that this feature was rarely observed in non-diabetic capillaries but was prominent in diabetic capillaries, with endothelial cells and pericytes being particularly susceptible to detachment (Fig. 1m, n). Although detachment of macroglial cells from the outer vascular BM was less common, in regions where it occurred, the cells appeared to retract from the capillaries (Fig. 1o, p and ESM Fig. 2).

### Endothelial tubules

**Fig. 2:**
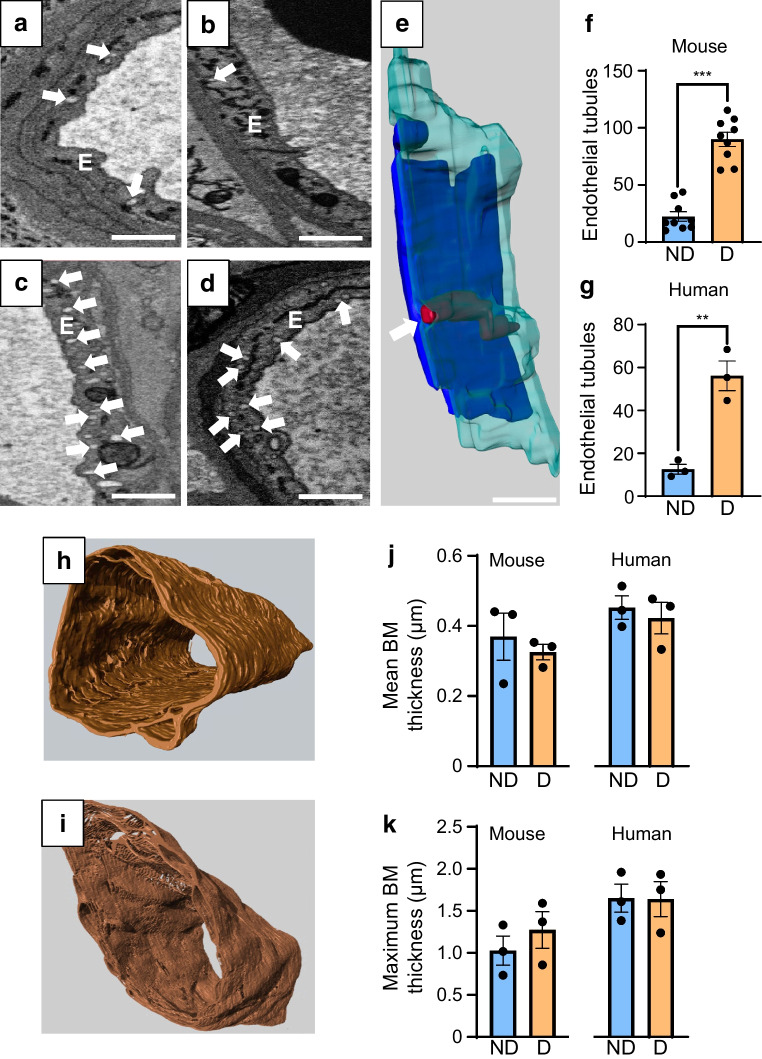
Endothelial tubule formation and vascular BM thickness in diabetic retinas. (**a**, **b**) Electron micrographs of retinal capillaries from a non-diabetic mouse (**a**) and an individual without diabetes (**b**), showing a small number of electron-lucent intracytoplasmic vesicles (white arrows) within endothelial cells. Scale bars, 1 µm. (**c**, **d**) Electron micrographs of retinal capillaries from a diabetic mouse (**c**) and an individual with diabetes (**d**), revealing a marked increase in intracytoplasmic vesicle formation. Scale bars, 1 µm. (**e**) 3D reconstruction of a diabetic mouse capillary showing a vesicle-derived tubular structure. Pericyte shown in blue, endothelium in aqua and tubule in red. (**f**, **g**) Quantification of endothelial tubules per 10 µm retinal capillary depth in non-diabetic and diabetic mice (*n*=3 mice, nine capillaries per group) and human donors (*n*=1 donor, three capillaries per group). **p<0.01, ***p<0.001. (**h**, **i**) 3D reconstructions of the vascular BM from a non-diabetic (**h**) and diabetic (**i**) mouse retina, showing no obvious thickening of the BM in diabetes. (**j**, **k**) Quantification of mean and maximum BM thickness in non-diabetic and diabetic capillaries from both mouse (*n*=3 mice, three capillaries per group) and human (*n*=1 donor, three capillaries per group) samples, showing no significant differences between the groups. D, diabetes; E, endothelial cells; ND, no diabetes

Several TEM-based studies have reported an increase in intracytoplasmic vesicles within retinal endothelial cells during experimental and human diabetes [6]. These vesicles are thought to contribute to increased transcellular permeability and breakdown of the inner blood–retina barrier (iBRB) in DRD. However, their precise 3D structure has remained unclear and previous suggestions that they may form transendothelial tunnels across the retinal endothelium [7] have yet to be thoroughly investigated. 3D reconstruction of endothelial vesicles in non-diabetic and diabetic mouse and human retinas revealed that these vesicles form tubular structures (Fig. 2a–e). Analysis of >1500 tubules from all capillaries examined showed that while some occasionally had openings on the luminal or abluminal side of the endothelium, none formed complete transendothelial channels (Fig. 2e). As expected, diabetic endothelial cells exhibited a significant increase in the number of endothelial tubules when compared with non-diabetic counterparts (Fig. 2f, g).

### Vascular BM thicknesses

Vascular BM thickening is traditionally considered one of the earliest ultrastructural changes in DRD [8]. To determine whether the pathological changes observed in peg-and-socket formations, cell–BM interactions and endothelial tubule formation coincide with BM thickening, we developed a MATLAB script to automate the measurement of mean and maximum vascular BM thickness across the 2D image stacks of the retinal NVUs. As shown in Fig. 2h–k and ESM Figs 3 and 4, no significant differences were found in mean or maximum BM thickness when comparing non-diabetic with diabetic mouse or human retinal capillaries analysed in this study.

## Discussion

In this study, we employed SBF-SEM to examine 3D ultrastructural changes in the retinal NVU of diabetic mice and humans, uncovering novel pathological features not previously reported in the literature.

A key observation was the partial detachment and reduced frequency of pericyte–endothelial cell peg-and-socket formations in the diabetic retina. This disruption in pericyte–endothelial cell adhesion may destabilise the capillaries, contribute to the breakdown of the iBRB and impair intercellular communication between these cell types. Notably, previous studies have highlighted that peg-and-socket formations serve as critical sites for the enrichment of angiopoietin-1 (ANG-1)–tyrosine kinase with immunoglobulin and epidermal growth factor homology domain-2 (TIE2) signalling in retinal capillaries [5]. This pathway is vital for maintaining endothelial junction integrity, promoting endothelial cell survival and reducing endothelial inflammation [9]. Disruption of these formations in DRD may therefore impair ANG-1–TIE2 signalling, exacerbating endothelial dysfunction, vascular leakage and inflammation, which are known to accelerate the progression of DRD [1].

We also observed local cell detachment from the vascular BM in diabetic capillaries, particularly affecting the endothelial cells and pericytes. Beyond peg-and-socket disruption, this detachment may further exacerbate vascular instability and contribute to the apoptosis of these cells in DRD, as cell adhesion is essential for their survival [1]. Additionally, given the well-established role of macroglial–pericyte signalling in neurovascular coupling in the retina [10], the retraction of macroglial cells from the outer vascular BM could underlie the disruption of this process in DRD, resulting in a mismatch between retinal capillary blood flow and the metabolic demands of retinal neurons.

The increased formation of endothelial tubules in diabetic retinas was another notable finding. While we found no evidence that these form complete transendothelial channels, limitations in section thickness in our SBF-SEM imaging (100 nm) prevented us from fully ruling this out. Techniques such as focused ion beam-SEM, offering better z-resolution (5–20 nm), could help validate these findings. The exact contribution of these tubules to vascular leakage, iBRB disruption and oedema formation in DRD remains to be determined but their increased frequency in diabetic retinas warrants further investigation.

A limitation of the present study is that 3D ultrastructural analysis using SBF-SEM was confined to capillary regions within the superficial vascular plexus. Recent SBF-SEM studies in murine and primate retinas have shown that NVU ultrastructural architecture differs considerably between the superficial, intermediate and deep vascular plexuses [3, 11]. As such, future investigations assessing whether similar abnormalities occur in the intermediate and deep capillary networks will be important to fully characterise NVU dysfunction across the entire retinal vasculature in DRD. Additionally, this study included only male mice and human donors, which may limit the generalisability of the findings. Given reported sex differences in DRD [12], caution is warranted when extrapolating these results to the female sex, highlighting the need for further research including both sexes.

Taken together, these findings highlight the value of examining the 3D architecture and cellular interactions within the retinal NVU to gain clearer insight into early pathological events in DRD. Interestingly, the novel ultrastructural changes we have identified seem to occur without overt vascular BM thickening, suggesting that these pathologies may develop independently and emerge before BM thickening becomes detectable. Further studies are now needed to determine the functional consequences of these structural abnormalities and clarify their contribution to NVU dysfunction and disease progression in DRD.

## Supplementary Information

Below is the link to the electronic supplementary material.

ESM (PDF 4.16 MB)

## Data Availability

Links to all raw image stacks analysed in this article are available at 10.5281/zenodo.15210333. The MATLAB vascular BM thickness measurement script is available at the GitHub link https://github.com/Curtis-WWIEM/BM_thickness.

## Abbreviations

ANG-1: Angiopoietin-1
BM: Basement membrane
DRD: Diabetic retinal disease
iBRB: Inner blood–retina barrier
NVU: Neurovascular unit
SBF-SEM: Serial block-face scanning electron microscopy
SEM: Scanning electron microscopy
STZ: Streptozocin
TEM: Transmission electron microscopy
TIE2: Tyrosine kinase with immunoglobulin and epidermal growth factor homology domain-2
