# Supplementary material for: 3D electron microscopy reveals novel ultrastructural changes in the diabetic retinal neurovascular unit
Source: Diabetologia. 2025 Oct 2;68(12):2899–905. doi: 10.1007/s00125-025-06554-9 (PMC12594679; doi:10.1007/s00125-025-06554-9)
Supplement: Supplementary file 1 — ESM (PDF 4.16 MB) [file 125_2025_6554_MOESM1_ESM.pdf]

3D electron microscopy reveals novel ultrastructural changes in the diabetic retinal neurovascular unit

ESM Methods

Diabetic mouse samples

All animal work was approved by the Queen's University Belfast Animal Welfare and Ethical Review Body (AWERB) and conducted in accordance with the UK Animals (Scientific Procedures) Act 1986 under DHSSPS/Home Office project licence PPL2919. Male C57BL/6J mice, bred in-house, were selected to reduce variability and because female mice exhibit significantly greater resistance to the diabetogenic effects of streptozocin compared to males. These mice were randomly allocated to non-diabetic or diabetic groups using a coin toss. Mice in the diabetic group were injected intraperitoneally with streptozocin (STZ, Sigma, Gillingham, UK catalogue #S0130) dissolved in sodium citrate buffer (pH 4.5) at 6–8 weeks of age. A dose of 50 mg/kg body weight was administered once daily for five consecutive days. During this period, mice received 10% (w/v) sucrose in water and were housed at 26°C under a 12-hour light/dark cycle with ad libitum access to food and water. Control mice, assigned to the non-diabetic group, were injected with citrate buffer only and co-housed with STZ-treated mice. One week after the final STZ injection, non-fasting venous blood glucose levels were measured using an SD Codefree glucometer (SD Biosensor Inc., Republic of Korea) to confirm diabetic status, defined as blood glucose >18 mmol/l. Mouse weights were monitored 1 to 2 times per week. After six months of diabetes, mice were euthanised by CO_2 asphyxiation and cervical dislocation, weighed, and blood samples were collected for HbA_{1c} measurement (BHR Pharmaceuticals, Nuneaton, UK; catalogue# HB-3058). Characteristics of the non-diabetic and diabetic mice are provided in ESM Table 1.

Diabetic human samples

Human eye samples were obtained with written informed consent, approved by the Newcastle University Research Ethics Committee (17/NE/0361), and the study was conducted in accordance with the Declaration of Helsinki. The eyes were collected from patients undergoing exenteration for large facial/sinus tumours at the Royal Victoria Infirmary, Newcastle upon Tyne. Immediately after collection, fixative (2% glutaraldehyde in 0.1 M sodium cacodylate

buffer, pH 7.3) was injected into the eyes. Two human eyes were examined: one from a 67-year-old male with a 10-year history of diabetes mellitus (type 2, managed with oral medication) and minimal background diabetic retinopathy without maculopathy (occasional peripheral microaneurysms only); and the other from a 58-year-old male with no history of diabetes and normal blood glucose.

Sample preparation

Mouse and human retinas were microdissected and immersed in fixative (2% glutaraldehyde in 0.1 M sodium cacodylate buffer, pH 7.3) for at least 12 hours. The human retina area used was approximately 1-1.5mm temporal from the foveal centre. The samples were subsequently processed with a heavy metal staining protocol to create an electron-dense surface for interaction with the electron beam[1, 2]. The samples were then embedded in resin (Taab 812 epoxy resin) and left to polymerise for a minimum of 36 hours. The resin blocks were trimmed with a razor blade to create a trapezoidal block face, then further trimmed to approximately 0.75 mm × 0.75 mm and glued onto a pin.

Image collection

Resin-embedded retinal tissue samples were imaged using a Zeiss Sigma SEM Gatan 3View System (Zeiss, Cambridge, UK; Gatan, Abingdon, UK). The system was used to locate retinal capillaries within the superficial vascular plexus and maintain them in the centre of the field of view during image acquisition. Sections were cut using a diamond ultramicrotome set to 100 nm for both mouse and human samples. Between 130 and 300 consecutive micrographs were captured for each capillary. Image dimensions were set to 3000 × 3000 pixels, with a pixel size of 6 nm and a dwell time of 20 μs/pixel.

Image processing

DM3 files obtained from the SBF-SEM were imported into Microscopy Image Browser (https://mib.helsinki.fi/; University of Helsinki; MIB v2.1) for post-processing [3]. Image contrast was enhanced, and contrast normalisation was applied across the z-dimension for all images in the stack. The images were then aligned using the drift correction tool. To reduce dataset size and improve processing speed, the images were converted from 16-bit to 8-bit.

The cellular and vascular basement membrane (BM) components of the retinal NVU's were examined and identified based on their location within the capillary or their morphological characteristics[4]. Each feature of interest was segmented and color-coded throughout the z-stack data using a combination of manual segmentation and semi-automatic interpolation. The features were color-coded as follows: BM (brown), endothelial cells (aqua), endothelial tubules (red), and pericytes (blue). For 3D reconstruction, segmented files were exported as Amira mesh binary format (.am) and imported into Amira (2023.1.1) software (ThermoFisher, Loughborough, UK). No formal blinding procedures were implemented during image processing or outcome assessment in this study.

Image analysis

Criteria for defining specific NVU features

To quantify NVU features, we established the following criteria for identifying peg-and-socket formations, cell-BM detachment, and endothelial tubules in mouse and human retinal NVU image stacks:

a. Peg-and-socket formations

These features must:

1. Span a minimum distance of 200 nm in the z-dimension.
2. Contain at least one section where the peg attaches to the cell body.
3. Transverse the BM.
4. Be engulfed by a neighbouring cell.
5. Have a minimum transverse width of 0.07 μm and a minimum longitudinal length of 0.1 μm.

We refer to these structures as peg-and-socket 'formations' rather than 'junctions,' as commonly described in the literature[5]. This terminology is based on transmission electron microscopy studies (For methodological details, see [6]) examining 90 of these structures in 2D images, where we found no evidence of intercellular junctional complexes between the pegs and sockets (ESM Fig. 5a). This was despite inter-endothelial junctional complexes being readily identifiable in the same images (ESM Fig. 5b).

b. Endothelial-BM detachment

These features must:

1. Exhibit an electron lucent appearance.
2. Be observed in the endothelial-to-BM space.
3. Span a minimum depth of 200 nm in the z-dimension.
4. Have a minimum width of 20 nm.
5. Exclude areas of BM absence caused by peg and socket formations or pericyte coverage.
6. Display no breaks or interruptions in the continuity of separation over at least 200 nm.

c. Pericyte-BM detachment

These features must:

1. Exhibit an electron lucent appearance.
2. Be present in the pericyte-to-BM space.
3. Span a minimum depth of 200 nm in the z-dimension.
4. Have a minimum width of 20 nm.
5. Show no breaks or interruptions in the continuity of separation over at least 200 nm.

d. Macroglia-outer BM detachment

These features must:

1. Exhibit an electron lucent appearance.
2. Be present between the macroglial-to-BM space.
3. Span a minimum depth of 200 nm in the z-dimension.
4. Have a minimum width of 20 nm.
5. Display no breaks or interruptions in the continuity of separation over at least 200 nm.

e. Endothelial tubules

These features must:

- Exhibit an electron-lucent appearance throughout their 3D depth.
- Not result from luminal endothelial microvilli arching on the endothelial surface.

- Span a minimum depth of 200 nm in the z-dimension.
- Have a minimum width of 60 nm.
- Maintain a rounded shape throughout their 3D depth, except where an opening is present on the luminal or abluminal side of the endothelial membrane.

Endothelial tubules were quantified regardless of their orientation within the endothelial cytoplasm.

Analysis of vascular BM thicknesses

MATLAB script and input data

The MATLAB (https://www.mathworks.com/products/matlab.html; MathWorks; R2020a) script for analysing vascular BM thickness in this study is available on GitHub (https://github.com/Curtis-WWIEM/BM_thickness). It requires the Image Processing Toolbox for full functionality. The input consisted of user-selected TIFF image stacks from the SBF-SEM datasets, with the BM segmented using Microscopy Image Browser (MIB v2.1) [3].

Binary segmentation of the BM

Specified slices in the image stack were loaded sequentially using “imread.” The script then generated a binary mask for the BM by collecting all segmented pixels in each slice and inverting them to facilitate distance transformation calculations.

Distance transformation and skeletonisation

A Euclidean distance transform was applied to the inverted binary mask using “bwdist.” This function computes the shortest distance from each BM pixel to the nearest non-BM pixel [7, 8]. The script then skeletonised the binary mask using “bwmorph” with the “thin” parameter, iteratively applying the Lam, Lee, and Suen (1992) algorithm [9] to morphologically erode the BM to a one-pixel-wide skeleton.

Thickness measurements

For each slice, the script then extracts thickness values along the skeleton by mapping skeleton pixels to corresponding distance transform values. These values are converted to nanometers using a predefined scale (1 pixel = 6 nm). The thickness values are stored in a

cell array for later statistical analysis, including the computation of mean and maximum thickness values for each slice.

BM thickness measurement approach

In this study, each SBF-SEM dataset was analysed by manually segmenting the BM every 1/10 slices. To accurately determine its maximum thickness, 10 consecutive sections were segmented at the thickest point in the stack. Given the time-intensive nature of the segmentation process, BM measurements in the mouse studies were limited to a single capillary from each of the three non-diabetic and diabetic mice investigated.

Statistics

Data are presented as mean ± SEM, with individual data points shown as symbols. Normality was assessed using the Shapiro-Wilk test, and statistical analysis was conducted using unpaired two-tailed Student's t-tests or Mann-Whitney U tests, with $p < 0.05$ considered statistically significant.

References

1. Cocks E, Taggart M, Rind FC, White K (2018) A guide to analysis and reconstruction of serial block face scanning electron microscopy data. J Microsc 270(2): 217-234. 10.1111/jmi.12676

2. Wilke SA, Antonios JK, Bushong EA, et al. (2013) Deconstructing complexity: serial block-face electron microscopic analysis of the hippocampal mossy fiber synapse. J Neurosci 33(2): 507-522. 10.1523/JNEUROSCI.1600-12.2013

3. Belevich I, Joensuu M, Kumar D, Vihinen H, Jokitalo E (2016) Microscopy Image Browser: A Platform for Segmentation and Analysis of Multidimensional Datasets. PLoS Biol 14(1): e1002340. 10.1371/journal.pbio.1002340

4. Albargothy MJ, Azizah NN, Stewart SL, et al. (2023) Investigation of heterocellular features of the mouse retinal neurovascular unit by 3D electron microscopy. J Anat 243(2): 245-257. 10.1111/joa.13721

5. Abdelazim H, Payne LB, Nolan K, et al. (2022) Pericyte heterogeneity identified by 3D ultrastructural analysis of the microvessel wall. Front Physiol 13: 1016382. 10.3389/fphys.2022.1016382

6. Albargothy MJ (2024) Three-dimensional mapping of the retinal neurovascular unit in health and diabetes. In: Biosciences Institute, Faculty of Medical Sciences. Published PhD thesis. Newcastle University, p 170

7. Rosenfeld A, Pfaltz JL (1966) Sequential Operations in Digital Picture Processing. Journal of the ACM (JACM) 13(4): 471-494

8. Maurer CR, Qi RS, Raghavan V (2003) A linear time algorithm for computing exact Euclidean distance transforms of binary images in arbitrary dimensions. Ieee T Pattern Anal 25(2): 265-270. Doi 10.1109/Tpami.2003.1177156

9. Lam L, Lee SW, Suen CY (1992) Thinning Methodologies - a Comprehensive Survey. Ieee T Pattern Anal 14(9): 869-885. Doi 10.1109/34.161346

ESM Table 1. Body weight and HbA_{1c} measurements in non-diabetic and diabetic mice.

Mouse	Start Weight (g)	End Weight (g)	HbA_{1c} (mmol/mol)	HbA_{1c} (%)
Non-diabetic	26.0	29.0	26	4.6
Non-diabetic	26.2	34.0	34	5.3
Non-diabetic	27.0	35.8	26	4.6
Diabetic	25.7	26.7	110	12.2
Diabetic	22.5	24.9	95	10.9
Diabetic	25.1	25.0	>120	>13.1

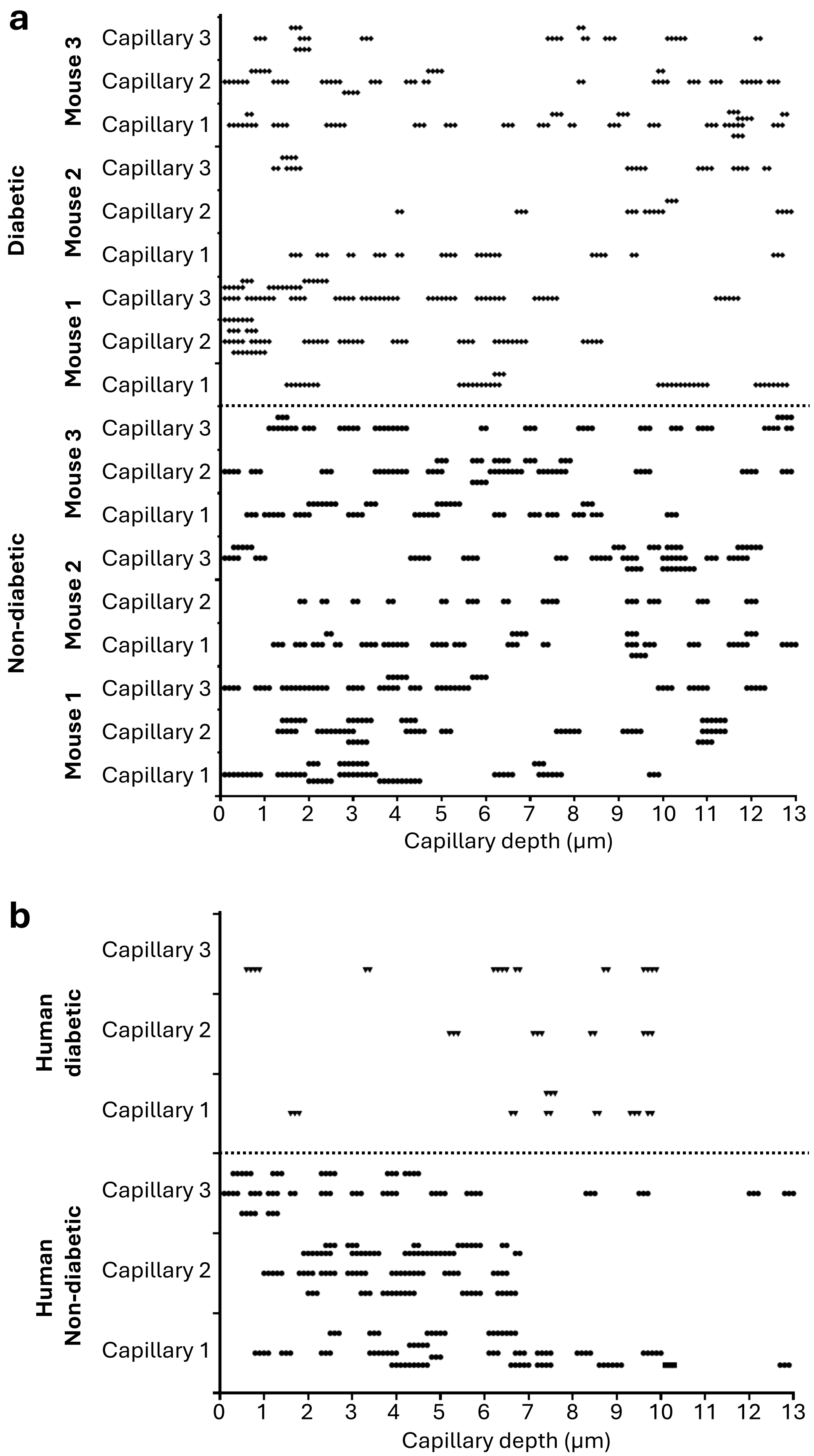

ESM Fig. 1. Distribution of peg-and-socket formations as a function of capillary depth in mouse (a) and human (b) non-diabetic and diabetic retinal capillaries. The occurrence of multiple peg-and-socket structures within the same section accounts for the multi-levelled appearance of individual capillary plots.

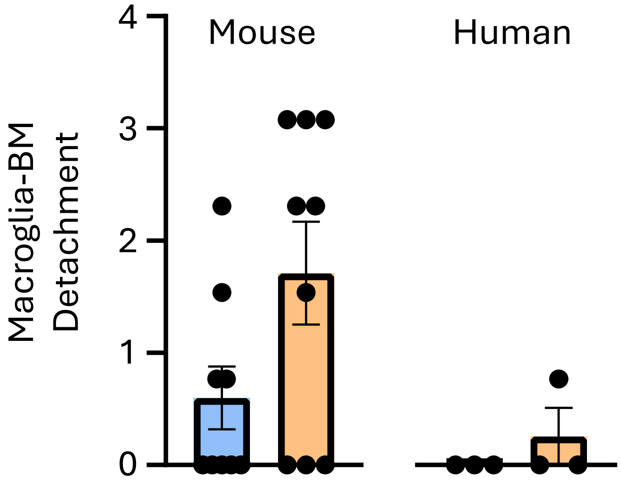

ESM Fig. 2. Quantification of macroglia detachment from the outer vascular BM per 10μm capillary depth in non-diabetic and diabetic mouse (n = 3 animals, 9 capillaries per group) and human (n = 1 donor, 3 capillaries per group) samples (blue bars, non-diabetic; orange bars, diabetic).

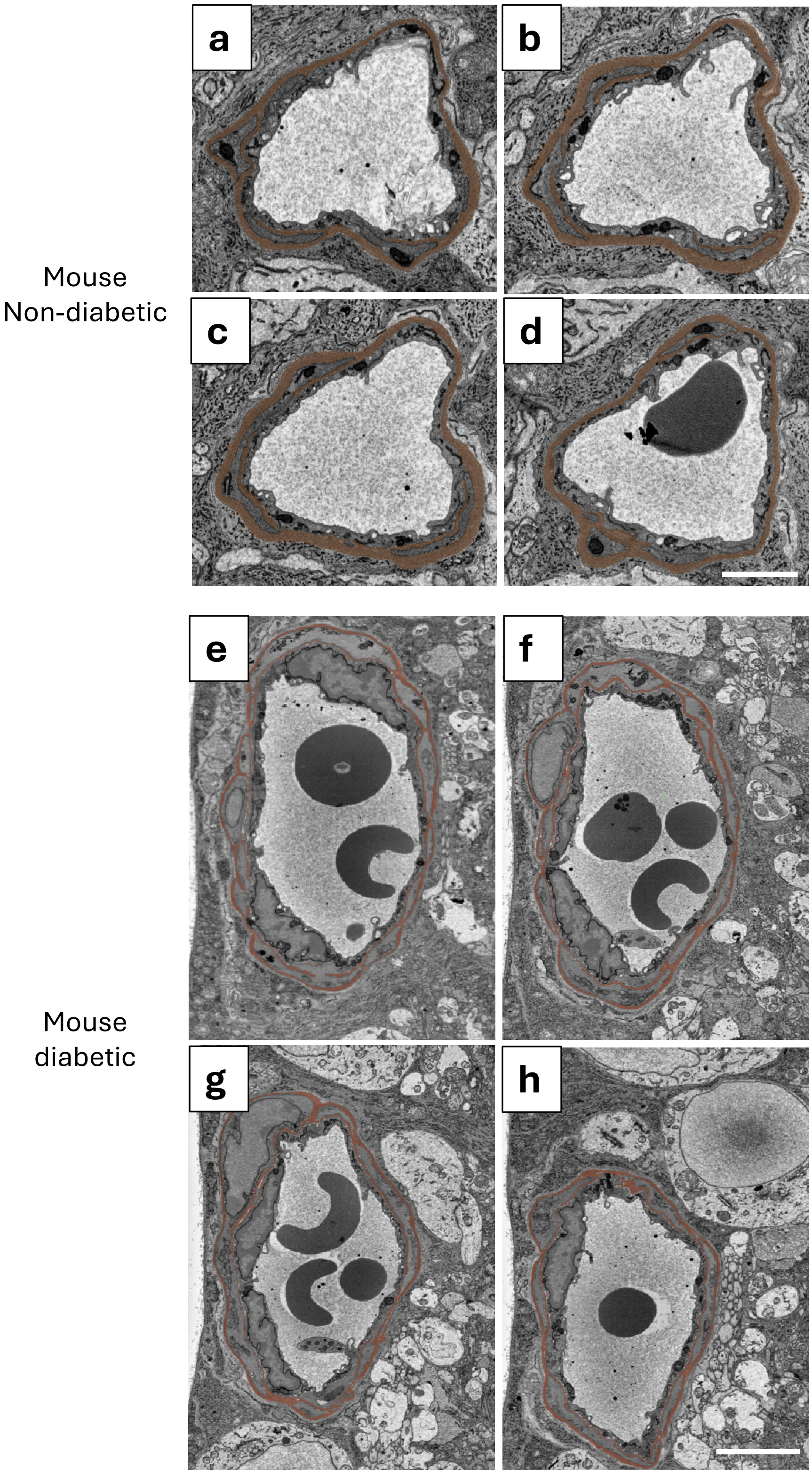

ESM Fig. 3. Representative images comparing BM thickness in retinal capillaries of non-diabetic and diabetic mice. (**a–d**) SBF-SEM micrographs of a non-diabetic mouse retinal capillary, with the BM segmented in brown at different depths: (a) slice 1 (0.1 μm), (b) slice 26 (2.6 μm), (c) slice 50 (5 μm), and (d) slice 100 (10 μm). (**e–h**) Corresponding images of a diabetic mouse retinal capillary at (e) slice 1 (0.1 μm), (f) slice 25 (2.5 μm), (g) slice 50 (5 μm), and (h) slice 90 (9 μm). These capillaries correspond to those shown in the 3D reconstructions in Fig. 2h, i. No clear visual evidence of BM thickening was observed in the diabetic retinal capillary. Scale bars: 4 μm.

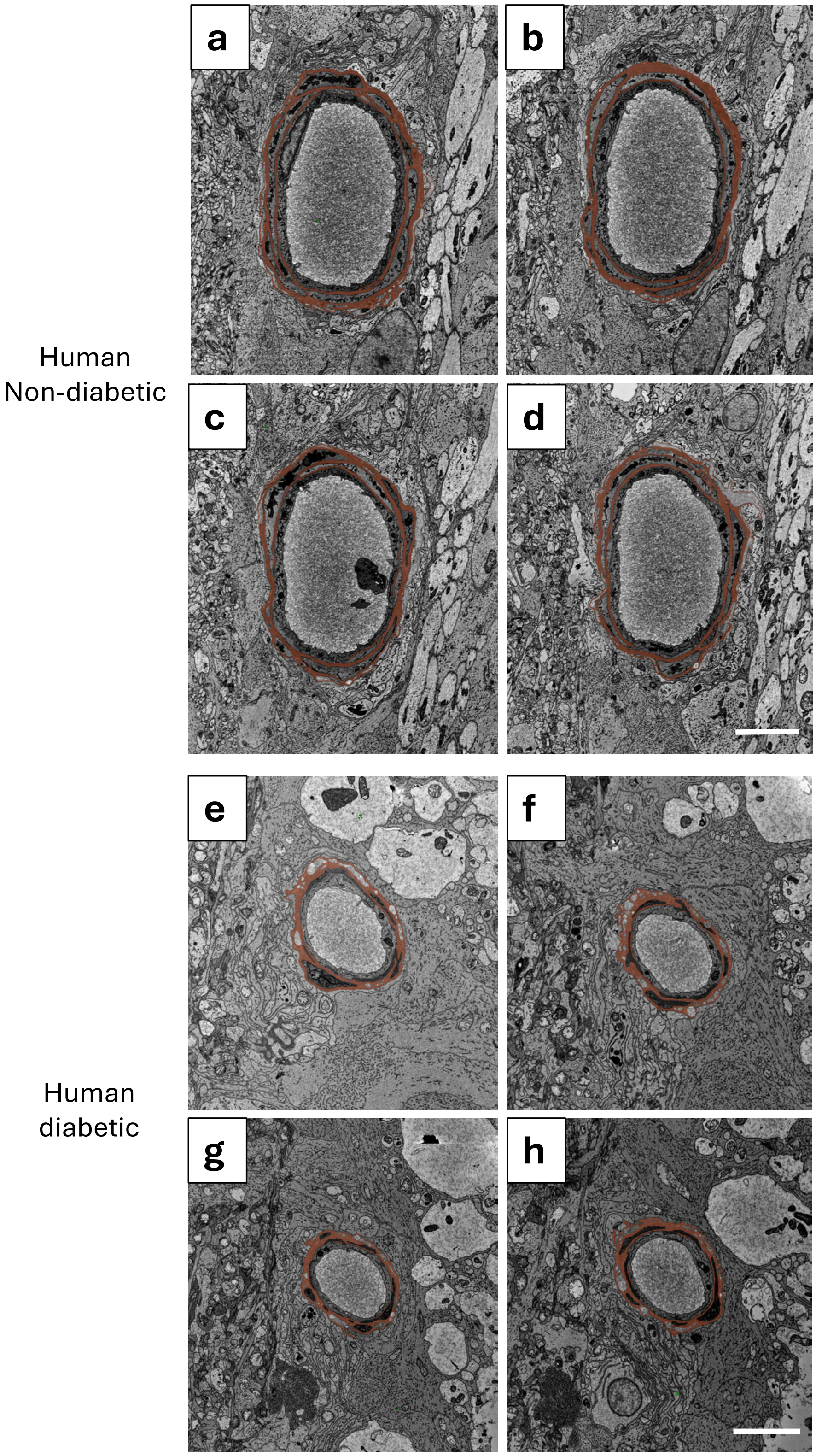

ESM Fig. 4. Representative images of BM thickness in retinal capillaries from non-diabetic and diabetic humans. (**a-d**) SBF-SEM micrographs of a non-diabetic human retinal capillary, showing the BM (segmented in brown) at different depths: (a) slice 1 (0.1 µm), (b) slice 30 (3 µm), (c) slice 60 (6 µm), and (d) slice 100 (10 µm). (**e–h**) Corresponding images of a diabetic human retinal capillary at (e) slice 1 (0.1 µm), (f) slice 30 (3 µm), (g) slice 60 (6 µm), and (h) slice 100 (10 µm). No clear visual evidence of BM thickening was observed in the diabetic retinal capillary. Scale bars: 4 µm.

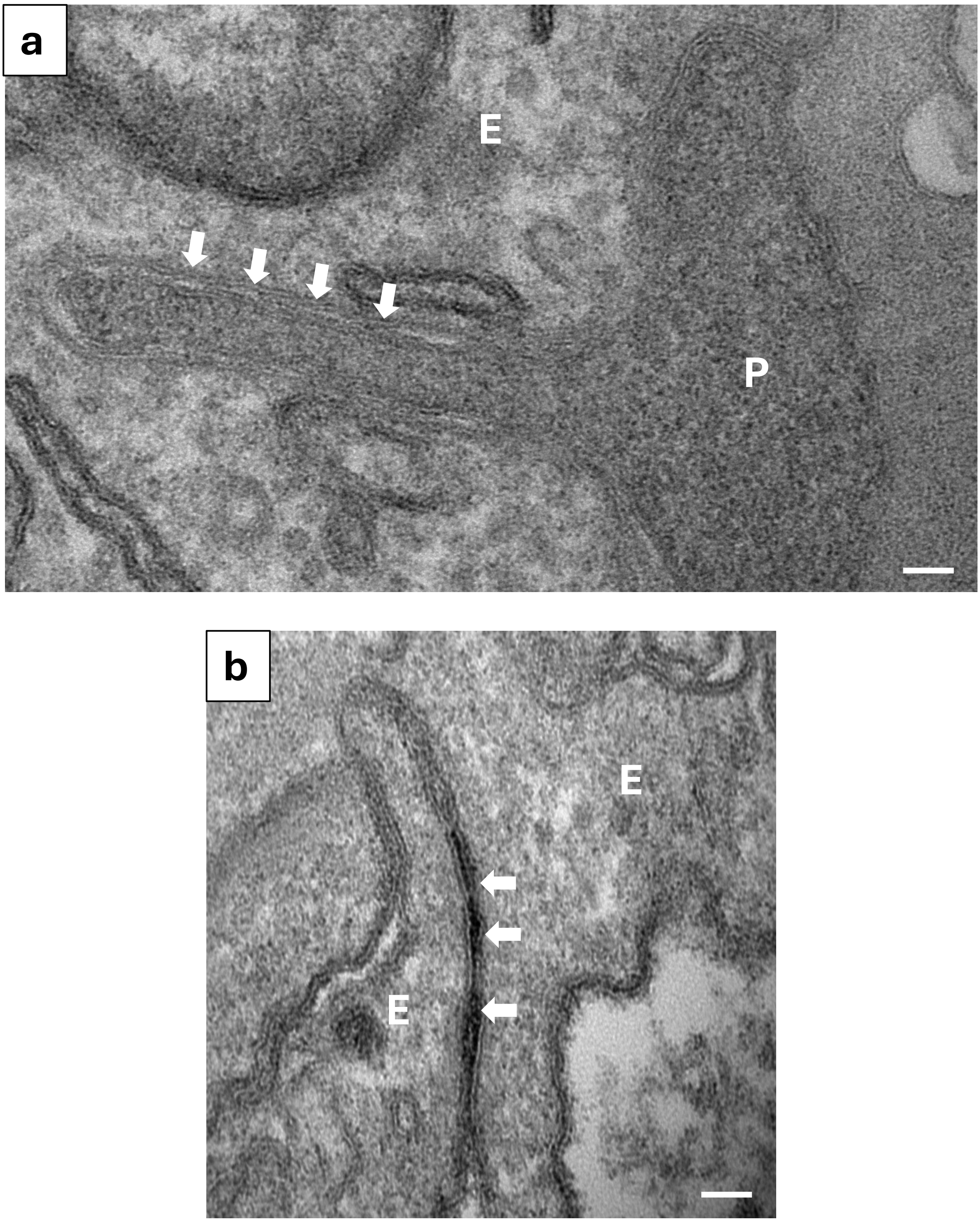

ESM Fig. 5. Transmission electron micrographs of a peg-and-socket formation and inter-endothelial junction in non-diabetic human retinal capillaries. (**a**) A peg-and-socket formation, highlighting structural details and the absence of clear junctions between the pericyte peg and endothelial socket membranes (white arrows). Scale bar: 0.2μm (**b**) While peg-and-sockets lacked evident junctions, inter-endothelial junctions (white arrows) were readily visible in the same image sets. Scale bar: 0.05μm. P, pericyte; E, endothelial cell.
